# Methodology and reporting quality of reporting guidelines: systematic review

**DOI:** 10.1186/s12874-015-0069-z

**Published:** 2015-09-22

**Authors:** Xiaoqin Wang, Yaolong Chen, Nan Yang, Wei Deng, Qi Wang, Nan Li, Liang Yao, Dang Wei, Gen Chen, Kehu Yang

**Affiliations:** Evidence-Based Medicine Center, School of Basic Medical Sciences, Lanzhou University, Lanzhou, 730000 China; Key Laboratory of Evidence Based Medicine and Knowledge Translation of Gansu Province, Lanzhou, 730000 China; Chinese GRADE Center, Lanzhou University, Lanzhou, 730000 China; Institute of Pathogenic Biology of Lanzhou University, Lanzhou, 730000 China; The First Hospital of Lanzhou University, Lanzhou, 730000 China; The Second Affiliated Hospital of Lanzhou University, Lanzhou, 730000 China

**Keywords:** Systematic review, Methodology quality, Reporting quality, Reporting guideline

## Abstract

**Background:**

With increasing attention put on the methodology of reporting guidelines, Moher et al. conducted a review of reporting guidelines up to December 2009. Information gaps appeared on many aspects. Therefore, in 2010, the Guidance for Developers of Health Research Reporting Guidelines was developed. With more than four years passed and a considerable investment was put into reporting guideline development, a large number of new, updated, and expanded reporting guidelines have become available since January 2010. We aimed to systematically review the reporting guidelines published since January 2010, and investigate the application of the Guidance.

**Methods:**

We systematically searched databases including the Cochrane Methodology Register, MEDLINE, and EMBASE, and retrieved EQUATOR and the website (if available) to find reporting guidelines as well as their accompanying documents. We screened the titles and abstracts resulting from searches and extracted data. We focused on the methodology and reporting of the included guidelines, and described information with a series of tables and narrative summaries. Data were summarized descriptively using frequencies, proportions, and medians as appropriate.

**Results:**

Twenty-eight and 32 reporting guidelines were retrieved from databases and EQUATOR network, respectively. Reporting guidelines were designed for a broad spectrum of types of research. A considerable number of reporting guidelines were published and updated in recent years. Methods of initial items were given in 45 (75 %) guidelines. Thirty-eight (63 %) guidelines reported they have reached consensus, and 35 (58 %) described their consensus methods. Only 9 (15 %) guidelines followed the Guidance.

**Conclusions:**

Only few guidelines were developed complying with the Guidance. More attention should be paid to the quality of reporting guidelines.

**Electronic supplementary material:**

The online version of this article (doi:10.1186/s12874-015-0069-z) contains supplementary material, which is available to authorized users.

## Background

The main purpose of investments for health research is to promote scientific understanding and improve health [1]. However, insufficient reporting of the methodology and findings of a study block critical appraisal and limit effective dissemination. In addition, insufficient reporting can also impede the applicability and mislead results used by patients and practitioners [2].

For improving the quality of the research, experts developed reporting guidelines. Developed with explicit methodology, a reporting guideline is a checklist, flow diagram, or an explicit text guiding authors in reporting a specific type of research. Some reporting guidelines provide a flow diagram for users to report information of research following sequential stages. Reporting guidelines are important tools for improving the quality of medical research. Since the development of the CONSORT (Consolidated Standards of Reporting Trials Statement for reporting randomized controlled trials) Statement in 1996, several guidelines have been developed relating to other types of research [2]. Guidelines such as CONSORT, PRISMA (Transparent reporting of systematic reviews and meta-analysis) [3], STARD (Standards for Reporting of Diagnostic Accuracy Studies) [4], STROBE (Strengthening the Reporting of Observational Studies in Epidemiology) [5], ARRIVE (Animal Research: Reporting: In Vivo Experiments Guidelines) [6], and COGS (The Conference on Guideline Standardization) [7] have largely benefited research in respect to RCTs (randomized controlled trials), systematic reviews and meta-analyses, animal research, and clinical practice guidelines. Adoption of reporting guidelines is associated with improved reporting quality of research [8]. Reporting guidelines can also act as handbooks for various people such as evidence developers and users, policy makers, editors, and patients, to play an optimistic role for regulating design and review of studies.

With increasing attention put on the methodology of reporting guidelines, Moher et al. [9] published *Guidance for Developers of Health Research Reporting Guidelines* (hereinafter refer to as “the Guidance”) in 2010, and conducted a review of 81 reporting guidelines up to December 2009 [10]. This review focused on guidelines using consensus-based methods and implemented deep analyses with the consensus method, where information gaps appeared within many aspects. For example, 28 % of the included guidelines reported no information about consensus, and 57 % were silent about how the feedback after consensus was dealt with. However, many of the reporting guidelines have not reported the implementation plan and pilot test. Insufficient reporting of the reporting guidelines enterprise could lead to inappropriate understanding and use. In addition to the methodology, only 31 % reported formal consensus method and 10 % applied the modified Delphi process [11], which indicated the methodology need to be improved. With considerable investment in the development of reporting guidelines, a large number of new, updated, and expanded reporting guidelines have become available since 2010. The number of reporting guidelines in the EQUATOR (Enhancing the QUAlity and Transparency Of health Research: www.equator-network.org) Network’s Library has increased from over 90 in December 2009 to over 200 in April 2014 on [12] and the EQUATOR also recommended the Guidance. Therefore, it is necessary to systematically review the reporting guidelines published since January 2010, learn the status of reporting and methodology quality, and investigate the application of the Guidance [9], so that we can know how well the improvement is achieved as well as how well the Guidance is applied.

## Methods

### Search strategies for identification of studies

We aimed to conduct a similar search strategy to Moher et al. After reviewing their search strategy and consulting with a librarian, we decided to search Cochrane Methodology Register, MEDLINE, and EMBASE databases. Unlike Moher et al., we did not search the PSYCInfo database, because it mainly includes articles from journals about the field of psychology, and we did not expect to find a large number of methodologically relevant articles. We referred to the search methods of Moher et al. [10], and used keywords such as “guideline”, “study design” in our search to identifying relevant evidence.

We made the following searches:

- Systematic search of the Cochrane Methodology Register, MEDLINE, and EMBASE.
- EQUATOR Library for health research reporting (www.equator-network.org).
- Supplementary search of the website of each guideline (if available) to collect their accompanying documents including checklists, flowcharts, and explanation and elaboration (E&E).

The details of our search strategies are shown in Additional file 1.

### Inclusion and exclusion criteria

We retrieved reporting guidelines for various fields of medical research. We only included the most recent versions and accompanying documents, including checklists, flowcharts, and explanation and elaboration (E&E) documents. We excluded the following types of guidelines: 1) how to report a specific research project, such as a guide for conducting a questionnaire survey; 2) instructions for authors; 3) guidelines for reporting diagnostic, treatment and prognostic information by clinical practitioners, such the results of imaging and pathological findings; 4) guidelines published before December 2009.

### Identification of relevant studies

Pairs of review authors independently checked the titles and abstracts resulting from searches. All reviewers participated in the decision on whether a study should be included. The guidelines were identified independently by two reviewers (XQW and QW) and disagreements were resolved by involving a third reviewer (QFW). At this stage, we only excluded those classified as ‘exclude’ by both reviewers. Then, we used a form developed to document the process.

### Measured outcomes

We collected the following outcomes from each included paper:

1. Basic information such as title, short name, specific website, year of publication, language, authors and reference list.
2. Summary statistics for the reporting outcomes of main interest in relevant reporting guidelines, including background information (version, scope and target research type, users), pre-meeting activities (member identification and recruitment, recruit methods, pre-planned endpoint of consensus process, inclusion criteria of items), the face-to-face consensus meeting (the format of meeting, number and backgrounds of experts, meeting agenda and materials), post-meeting activities (pilot test and feedback collection), other information (checklist, flow diagram, E&E documents, endorsement, translations, implementation plan, research gap, conflicts of interests (COIs)).

### Data extraction

We designed our extraction protocol based on items from the Guidance and other relevant articles about reporting guidelines. Pairs of review authors (XQW and NL, QFW and CLW) independently performed the data extraction with a pre-designed extraction form which was pilot tested by all data extractors and modified accordingly before formally use. The review authors resolved discrepancies on the data extraction through discussion. A third review author (QW) was consulted to settle discrepancies.

We focused on the methodology and reporting quality of included guidelines and extracted the following data: 1) characteristics of the study including title, language, year of publication, authors, and guideline version, 2) background information including time-consumption, research type, reporting scope, and target users, 3) whether the consensus was reported and whether consensus meeting was held and its relevant activities provided, 4) post-publication activities including implementation, evaluation, and endorsement, 5) whether the guidelines were compliant with the Guidance (this was judged by searching the text for a declaration of following the Guidance, and by checking the reference list), and 6) other information including research gaps, limitations, funding, and COIs. If the consensus was reported, we also collected ① pre-consensus activities including candidate items generation, ② consensus activities including which consensus method was used and how the authors proceeded, and ③ post-consensus activities including pilot test and feedback collection. Where the information was unclear, we contacted the authors to confirm it.

### Data analysis

We described information with a series of tables and narrative summaries. Data was descriptively summarized using frequencies, proportions, and medians as appropriate. We then explored items (especially the core items, including “7 Prepare for the face-to-face meeting; 8 Present and discuss results of pre-meeting activities and relevant evidence; 8.1 Discuss the rationale for including items in the checklist; 8.3 Discuss strategy for producing documents; identify who will be involved in which activities; discuss authorship; 9 Develop the guidance statement; 12 Seek and deal with feedback and criticism; 13 Encourage guideline endorsement” in the reporting guidelines that referred to Guidance, and conducted a comparison between guidelines refer to the Guidance with those that did not. We reported our study according to the PRISMA statement [3].

This study was not registered in advance because of its scope limitation, but it was presented in the 3rd International Society for Evidence-Based Health Care Conference (ISEHC) 2014 in Taipei, Taiwan, November 2014, as an oral presentation (OS05-03).

## Results

**Fig. 1:**
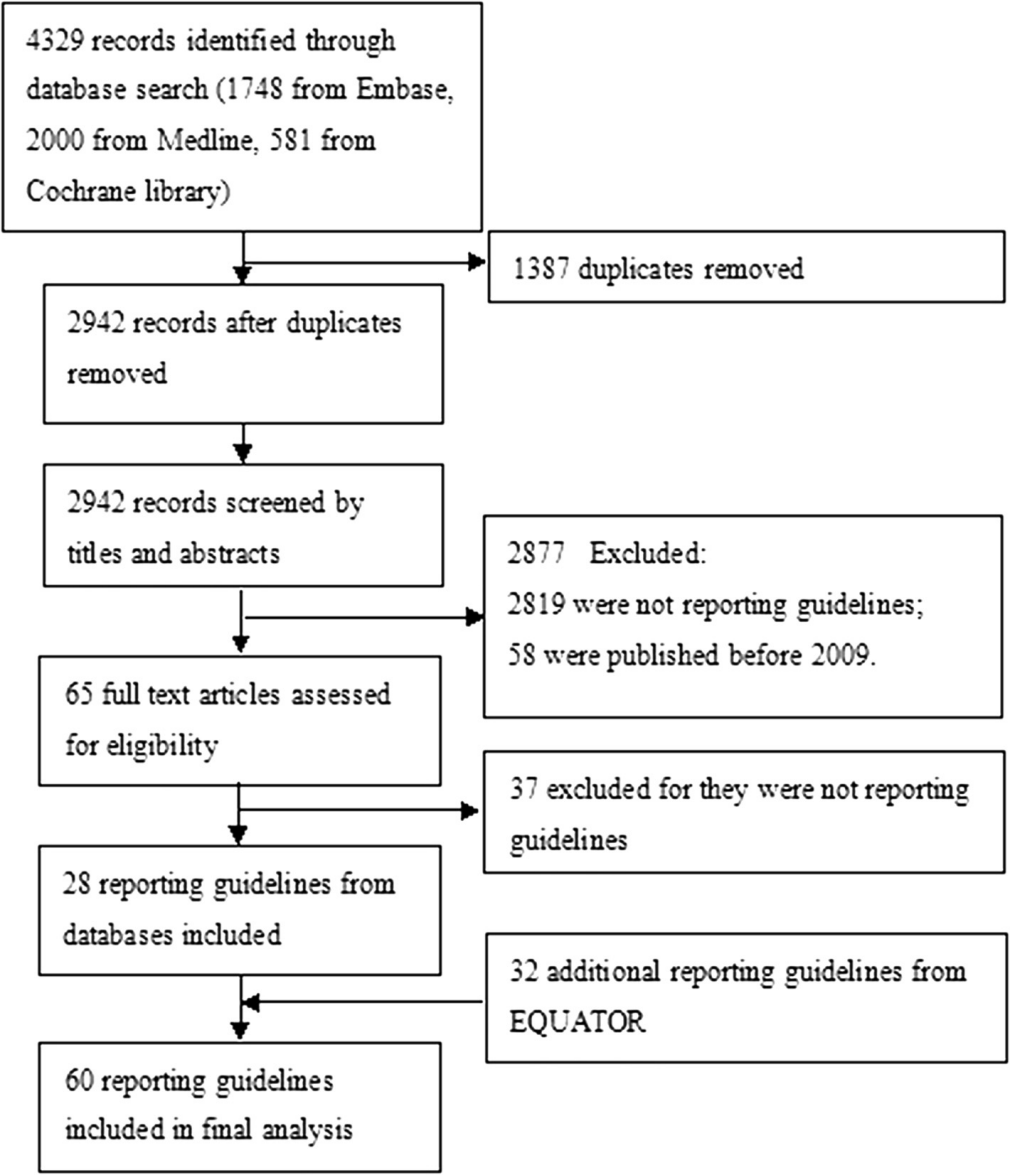
Flow chart of reporting guidelines identified, included and excluded. A total of 4329 records were hit by searching electronic databases. After 1387 duplicates removed, 2942 records were screened trough titles and abstracts, and 2877 were excluded. Then 65 full text articles assessed for eligibility, in which 37 were excluded. At last, together with 32 additional reporting guidelines from EQUATOR, 60 reporting guidelines were included

We identified 4329 records after screening the electronic databases. Of these, 28 full-text articles were retrieved. In addition, we found 32 relevant guidelines from the EQUATOR network that were not captured from databases. In total, 60 reporting guidelines for health research were included and were all used for data extraction (Fig. 1). Detailed information of the included reporting guidelines was presented in Additional file 2. We contacted the corresponding authors of five publications, of whom four helped us to confirm the information, and one gave no response.

### Characteristics of reporting guidelines

The basic information of the reporting guidelines is presented in Table 1. Reporting guidelines were designed for a broad spectrum of research typologies. A considerable number of reporting guidelines were published and updated and all of them were initially published in English.

**Table 1:** Basic information of included reporting guidelines

Items	Option	Number (%)
Short name	Provided	30 (50)
Specific website	Provided	14 (23)
Year of publication	2010	16 (28)
	2011	14 (23)
	2012	13 (22)
	2013	15 (25)
	2014	2 (3)
Development duration (year)	2	1 (2)
	3	1 (2)
	Not provided	58 (96)
Language of publication	English	60 (100)
Specific group	Provided	23 (38)
Information of develop members	Provided	59 (98)
Reference list	Provided	60 (100)

### Background of reporting guidelines

Thirty-eight (63 %) reporting guidelines were classified as new guidelines, 17 (28 %) were extensions of previously published guidelines, three (5 %) were updates of previous guidelines, and two (3 %) were updates of extensions. Two (5 %) of the new guidelines declared there will be extensions, and eight (13 %) of the extensions were proposed to be used along with other reporting guidelines, such as the original one. Thirty-nine (65 %) guidelines reported their target users, and nine (15 %) referred to the Guidance [9].

The scope of different reporting guidelines varied a lot. Thirty-six (60 %) were designed for full text, three (5 %) were used for reporting the methods, two (3 %) were developed for methods and discussion, and two (3 %) were designed for abstract, whereas, the remaining 17 (28 %) were unclear.

Forty (67 %) guidelines were designed for specific studies or multiple study designs. The most mentioned one catering to a specific design were about observational studies (11, 18 %) and RCTs (9, 15 %). Forty-four (73 %) reporting guidelines were designed for specific health areas.

Thirty-five (58 %) guidelines reviewed the literature and 22 (37 %) identified previous relevant guidelines. For search details, databases, terms, years, and languages were given in 12 (20 %), 10 (17 %), 11 (18 %) and seven (12 %) guidelines, respectively. Forty-five (75 %) guidelines described the methods used to generate initial items, including literature review, referring to existing reporting guidelines or opinions from experts (Table 2).

**Table 2:** Characteristics of reporting guidelines

Items	Items	Number (%)
Study type(s) to be reported^a^	Study design not specified	20 (33)
	Randomized controlled trials	9 (15)
	Laboratory/preclinical studies	5 (8)
	Prospective clinical trials	2 (3)
	Observational study^b^	11 (18)
	Economic evaluation	3 (5)
	Systematic review/meta-analysis/HTA^c^	5 (8)
	Qualitative research	2 (3)
	Cross-sectional studies	2 (3)
	Other specific design or types specified (diagnostic accuracy studies, quality improvement research, Realist syntheses, clinical practice guideline, Systematic review/meta-analysis/HTA)	12 (20)
Methods for initial items^d^	Literature review	25 (42)
	Refer to existing reporting guidelines	20 (33)
	Opinions of experts or consensus of experts	16 (27)
Consensus methods^e^		
Formal	Delphi	13 (22)
	Nominal group technique	3 (5)
	Consensus meeting	21 (35)
Informal	Questionnaire grade	1 (2)
	Circulating several versions of the statement within the group of developers and an external circle of potential users.	2 (3)
	Web-based survey rating the importance of each of the checklist items	1 (2)
Terminated after fixed round(s) of Delphi	1	2 (3)
	2	5 (8)
	3	4 (7)
Stakeholders in the consensus process	Content experts	25 (42)
	Epidemiologists	7 (12)
	Journal editors	14 (23)
	Methodologists	19 (32)
	Others^f^	14 (23)
Funding	Yes	36 (60)
	No	4 (7)
	Unclear	20 (33)
COI	Yes	14 (23)
	No	28 (47)
	Unclear	18 (30)

^a^Some reporting guidelines are designed for more than one type of research

^b^Including various kinds of observational studies, such as case report, cohort study, case–control study cross-sectional study

^c^HTA: Health technology assessment

^d^Some reporting guidelines used two or more methods to generate initial items

^e^Some reporting guidelines used two methods

^f^Including clinicians, funders, students, government agencies, professional organizations, publishers and patients

Funding was obtained to assist the development of at least 36 (60 %) reporting guidelines. This information was not reported or unclear in 20 (33 %).

### Pre-meeting activities

Thirty-eight (63 %) guidelines reported they have reached consensus and 35 (58 %) also described consensus methods, whereas no one gave the definition of consensus. For consensus methods, the formal methods was used in 23 (38 %) guidelines, in which eight (13 %) employed two methods. Delphi exercise was applied in 13 (22 %) guidelines. An informal method was identified in five (8 %) (Table 2).

Among guidelines developed through consensus, 30 (50 %) reported group member identification and 31 (52 %) reported member recruitment. Of those who identified members, 27 (45 %) reported specialties of experts, 20 (32 %) described information of members, such as names and institutions, and four (7 %) gave the selection criteria. For those who recruited members, seven (12 %) described the recruit methods, for instance, through e-mail, study co-chairs, or group decision. In guidelines developed by a working group, 22 (37 %) reported the number of experts participating in guideline development (median 32, range 3–115). Eleven (18 %) guidelines reported the endpoint of consensus process, which were all terminated after a fixed number of rounds (Table 2). In addition, the inclusion criteria of items were given in eight (13 %) guidelines. For example, items meeting the median score of eight or higher in the final round were included [13].

### The face-to-face consensus meeting

Consensus meeting was used in 24 guidelines. In 18 (30 %) guidelines only face to face meeting was held and in four (7 %) guidelines only a teleconference, and in two (3 %) guidelines, both face-to-face meeting and teleconference were held. Thirteen (22 %) guidelines reported the number of experts invited to a face-to-face meeting (median 23, range 9–51), and 15 (25 %) reported the number of experts attending the meeting in person (median 18, range 3–51). Five (8 %) reported developing a meeting agenda, nine (15 %) reported preparing materials that were sent to participants prior to meeting, and nine (15 %) detailed the duration of the meeting.

Guideline developers of the consensus process included stakeholders with various backgrounds (Table 2). The most commonly involved were content experts (25, 42 %), methodologists (19, 32 %), and journal editors (14, 23 %). However, activities of stakeholders for the development procedure and discussion during the consensus process were less commonly reported.

### Post-meeting activities

After the meeting held and the checklist completed, pilot tests were conducted in 12 (20 %) guidelines. Among these, 11 (18 %) described the pilot methods, seven (12 %) described the feedback information requirement, and five (8 %) gave the methods for feedback collection.

### Other information

The final items were presented in 58 (97 %) guidelines with a median of 22 (range: 5–106 items) items, and 44 (73 %) presented an explanation of each item. Items were summarized as a checklist in 54 (90 %) guidelines and seven (12 %) provided flow diagrams. Separate E&E documents were developed or under development in 16 (27 %) reporting guidelines. Eighteen (30 %) reported guideline endorsements with the mostly referred one as part of instruction to authors (13, 22 %). Publication strategies were reported in 12 (20 %) guidelines. Of those, 10 (17 %) were published in more than one journal with translations being (or to be) available for four (7 %) guidelines. The implementation and dissemination plan was found in 22 (37 %) guidelines, while information on monitoring and evaluation was given in 13 (22 %) guidelines. Sixteen (27 %) planned to deal with feedback after releasing the guidelines, and 15 (25 %) have updated or planned to update their guidelines. Four (7 %) guidelines elaborated the research gap and 21 (35 %) presented the limitations of reporting guidelines.

COIs were clearly reported in 42 (70 %) guidelines. Among these, 14 (23 %) declared they have COI, while 28 (47 %) did not give the information about COI (Table 2).

### Analysis of the core items from the guidance in reporting guidelines

**Fig. 2:**
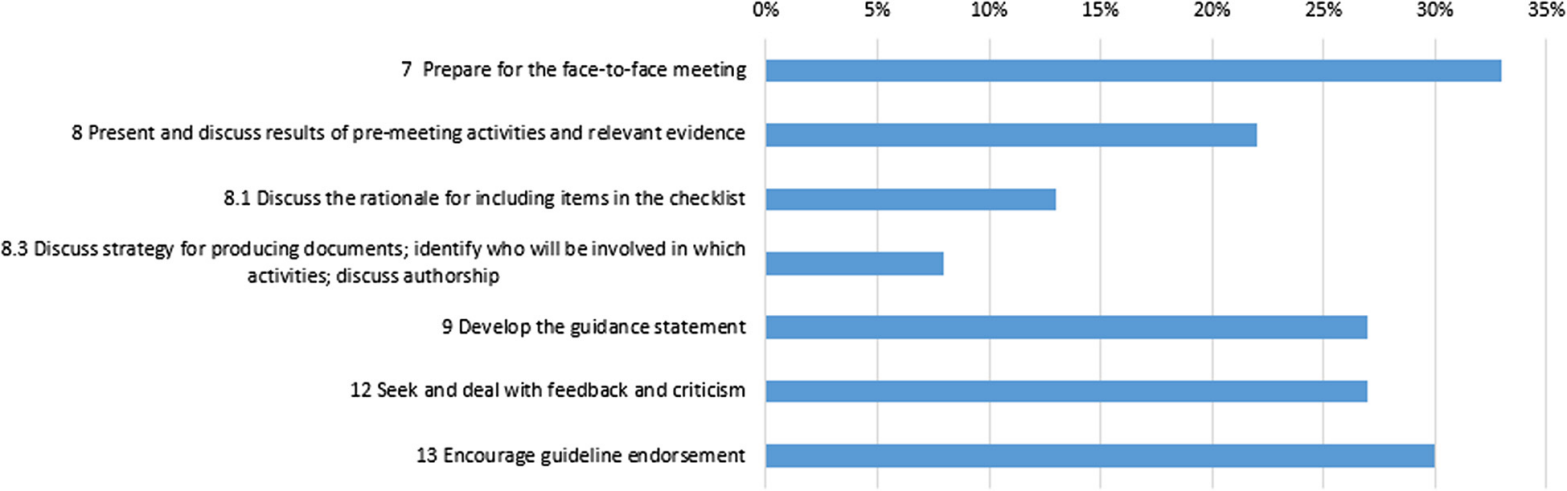
The information of core items in reporting guidelines

For the methodology of guideline development, we analyzed the core items from the guidance in the included reporting guidelines (Fig. 2). Which showed poor reporting in all the core items, every item was reported in less than 50 % reporting guidelines.

**Fig. 3:**
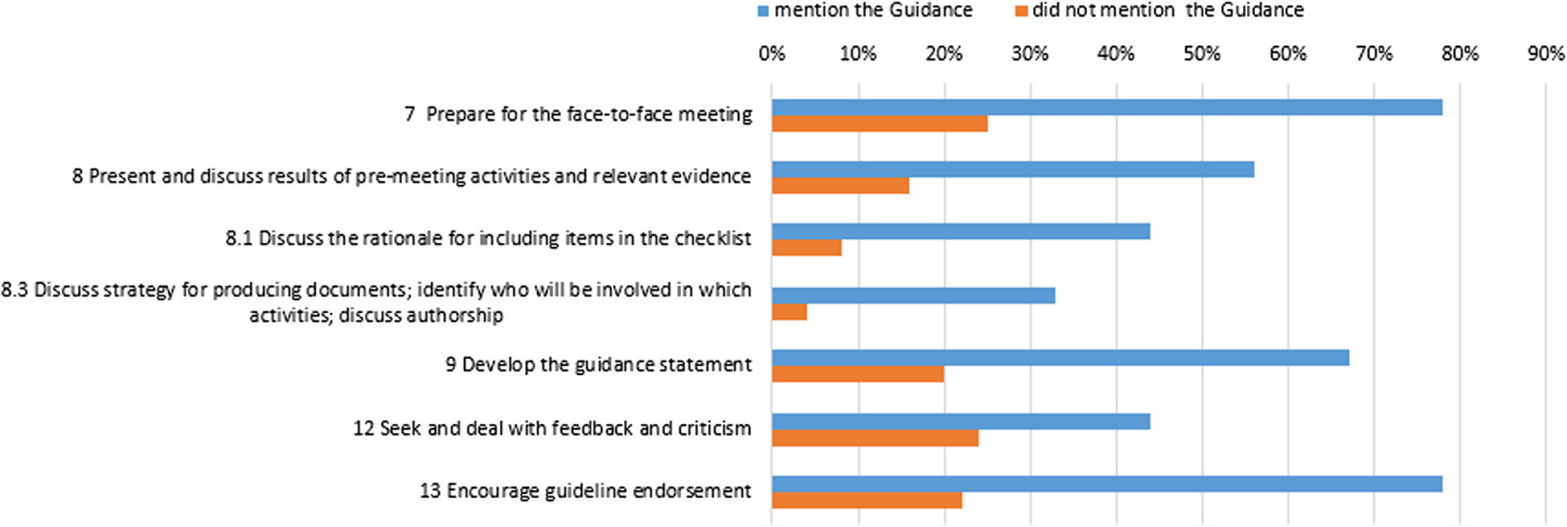
Comparison of guidelines that mentioned the Guidance vs those that did not

And then we conducted a comparison between the nine guidelines who said they have followed the Guidance [9] with those that did not regarding to the core items proposed in the Guidance (Fig. 3). Which indicated better conducting and reporting but still poor in some aspect, especially in “8.1 Discuss the rationale for including items in the checklist; 8.3 Discuss strategy for producing documents, identify who will be involved in which activities, discuss authorship; and 12 Seek and deal with feedback and criticism”.

The PRISMA checklist with the page number where each item is reported was provided in Additional file 3.

## Discussion

We identified 60 reporting guidelines for health research published since January 2010, most of which were new guidelines. Only few of the guidelines were developed in compliance with the Guidance [9]. More than half of the guidelines were developed for full texts and some for specific areas including methods, abstract, and discussion. However, a large portion did not state their target scope. Guidelines covered a broad spectrum of areas of health research. Previously, most guidelines were developed for RCTs [10], but now we found increasing attention was on other types of health research.

Only 37 % of the included guidelines searched the existing relevant reporting guidelines, and 58 % of the guidelines reviewed the literature. However, only few presented the search details. Since development of reporting guidelines is complex and time-consuming, developers should first consider whether a requirement exists for a new or extended reporting guidelines, or if the existing ones should continue to be used. Moreover, it is recommended to search for the relevant guidelines and evidence of the reporting quality of published articles of the domain of interest [9].

More than 30 % of the reporting guidelines did not report consensus. For those who did, details of consensus methods were poorly reported. These results were in line with the previous research [10]. Consensus methods were carried out both formally and informally. Formal consensus methods including Delphi, NGT (Nominal Group Technique), and Consensus development conference [14] were used. Consensus methods were used increasingly to solve problems in medicine and health areas. Their main purpose is to define levels of agreement on controversial subjects. Advocates suggested that, when properly employed, consensus strategies can create structured environments in which experts are given the best available information, thus, allowing solutions to problems to be more justifiable and credible than they would be otherwise [15]. What constituted a consensus should be provided in advance, together with the threshold specifying when consensus is reached [16]. Boulke et al. [17] recommended that Delphi rounds of two or three should be widely used when developing clinical guidelines [14] and reporting guidelines [9]. Consensus methods should be supported by developers, and the reporting of the methods should be improved.

Developers involved in the consensus process consisted of multidisciplinary teams of stakeholders, and most guideline development groups had the awareness about it. Among experts who participated in development, content experts were mostly referred, followed by methodologists and journal editors. This is in line with previous findings [10]. The expertise of developers should reflect the required skills of interest. Participants should include statisticians, epidemiologists, methodologists, content experts, journal editors, and consumer representatives. Furthermore, at least a quarter of the developers should be content experts, and this number could be even larger depending on content areas under consideration [9]. Ideally, invitations should be offered 6 months before the meeting. However, the detailed information on the participants, such as information about the members and the method applied for recruitment, was poorly reported.

Only few reporting guidelines tested the final checklist before formal publication, which was also found by Moher et al. [10]. As a small-scale trial, pilot testing provides a chance where a few examinees could take the test and comment on the object that was tested. They can point out any problems with the test instructions, instances where items are not clear, formatting, and other typographical errors and/or issues [18]. Once the information of pilot tests becomes available, the guideline group could decide whether to make changes to improve the product effectively.

Only a few reporting guidelines developed E&E documents [10], which were intended to enhance the use, understanding, and dissemination of the guidelines [19]. Developers are likely to publish guidelines in a simplified form to help use them effectively. Therefore, E&E documents are of critical importance to the rationale for including the items and the necessary details. Lack of this document may make it difficult to understand the guidelines. Additionally, the most important method for guideline dissemination was simultaneous publication of an E&E document [9].

Many of the reporting guidelines included did not give the implementation, evaluation, and monitoring strategies, which was similar with the previous finding [10]. Strategies for result sharing such as endorsement by organizations or journals, websites for reporting guidelines, and translations, have been recommended [9].

Although the reporting status was improved, the funding and COI were still not well reported. The proportion of reporting guidelines that acquired funding was also higher than before 2009 (60 vs 48 %) [10]. Conversely, the COI rules are designed to avoid potentially compromising situations that could affect, or otherwise undermine, the work by guideline group [20]. Although it is recommended that developers obtain funds from all sources including the pharmaceutical industry, non-profit agencies, and governments [9], they need to clearly state the funding sources and their role during the process of declaration of interests.

Except for the above content, even for the core items was not conducted well in the reporting guidelines. When it comes to the guidelines that mentioned the Guidance [9] vs those that did not, the results showed better results, which might suggest a positive effect of the Guidance. However, no matter followed the Guidance or not, some core aspects, including discussion on the rationale for including items, strategy for producing documents, and the feedback requirement, was still poorly conducted, which reminded us that there was still much work to do to improve the reporting and methodology of reporting guidelines.

More than 4 years after the systematic review conducted by Moher et al. [10], increasing numbers of reporting guidelines covering more and more study designs, such as animal research and case reports, were published. Unlike the previous review, we included reporting guidelines regardless of if the consensus were reached or not, in order to see the utilization of consensus process. This is the first research evaluating the application of the Guidance [9]. We did not limit the language when searching the reporting guidelines to minimize the undetected studies.

Our study also has some limitations. We did not search gray literature, which might miss some guidelines eligible for this review. But we encourage our readers to notify us if any possible eligible guidelines were missed. We abstracted data largely depending on the reporting information of guidelines, but we also asked the authors to confirm the unclear information. We mainly compared the core items proposed in the Guidance [9] between guidelines mentioned the Guidance vs those not, which could reflect the overall trend to some degree. We did not include all reporting guidelines listed on the EQUATOR because of the differences of the definition of reporting guidelines. For example, we excluded the Toolkit [21], which was included in EQUATOR, because it is not designed for reporting research.

## Conclusions

Increasing attention was paid to develop reporting guidelines other than for RCTs, including various observational studies and other types of study. Although some improvement was identified, details of consensus methods were also poorly reported, which were similar to previous findings [10]. From the comparison between guidelines which followed the Guidance with those did not, reporting guidelines mentioning the Guidance appeared to use more rigorous development methods, in line with the recommendations, than those that did not mention it. But only few guidelines were developed complying with the Guidance until now. Promoting the use of the Guidance might improve the development of reporting guidelines. The EQUATOR should also pay more attention to give some instructions for improving the quality of reporting guidelines as appropriate.

## Additional files

Additional file 1:
**Search strategies. **(PDF 75 kb) Additional file 2:
**Detailed information of the included reporting guidelines.** The information of the included reporting guidelines, including publication date, scope, focus, areas, version, and other relevant details. (PDF 161 kb) Additional file 3:
**The PRISMA checklist with the page number where each item is reported.** (PDF 204 kb)
