# Supplementary material for: Methodology and reporting quality of reporting guidelines: systematic review
Source: BMC Med Res Methodol. 2015 Sep 22;15:74. doi: 10.1186/s12874-015-0069-z (PMC4579604; doi:10.1186/s12874-015-0069-z)
Supplement: Additional file 1: — Search strategies. (PDF 75 kb) [file 12874_2015_69_MOESM1_ESM.pdf]

Additional file 1

MEDLINE

1. exp Research Design/
2. exp Guideline/
3. exp Study characteristics/
4. exp Epidemiologic studies/
5. Feasibility Studies/
6. Intervention Studies/
7. Program Evaluation/
8. Evidence-Based Medicine/
9. Human Experimentation/
10. exp Research/
11. Case Report/
12. Meta-analysis/
13. (case adj2 (report$ or series or control$)).mp.
14. ((control$ or clinical or comparative$) adj2 (trial$ or stud$)).mp.
15. between group design$.mp.
16. random$.tw.
17. ((control$ or intervention or evaluation or comparative or effectiveness or evaluation or feasibility) adj3 (trial or studies or study or program or design)).tw.
18. systematic review$.tw.
19. (time adj series).tw.
20. (pre test or pretest or posttest or post test).tw.
21. controlled before.tw.
22. (n-of-1 or single subject).tw.
23. or/1e22
24. (reporting and (trial$ or studies)).ti.
25. ((guideline$ or guide line$ or checklist$ or recommendation$ or standard$ or requirement$ or instruction$ or guidance$ or policies or policy) adj3 (reporting or publishing or good practice or good practi#e$)).tw.
26. or/24-25
27. (good adj3 practi#e$ adj3 (reporting or publishing or publication)).tw
28. (reporting adj2 (guideline or standard or standards)).ti.
29. (23 and 26) or 27 or 28
30. limit 29 to (english or french)

EMBASE

1. exp Research/
2. Epidemiology/
3. Randomized Controlled Trial/
4. exp Clinical Trial/
5. Meta Analysis/
6. Systematic Review/
7. Evidence Based Medicine/

8. Practice Guideline/
9. or/1e8
10. (reporting and (trial$ or study or studies)).ti.
11. ((guideline$ or guide line$ or checklist$ or check list$ or recommendation$ or standard$ or requirement$ or instruction$ or guidance$ or policies or policy) adj3 (reporting or publishing or good practice or good practi#e$)).tw.
12. (or/10e11) and 9
13. (reporting adj2 (guideline or standard or standards)).ti.
14. (good adj3 practi#e$ adj3 (reporting or publishing or publication)).tw.
15. or/12e14
16. limit 15 to (english or french)

CMR eWiley

Record title: (reporting near/2 (guideline or standard or standards))

OR

Search all text: ((guideline* or checklist* or recommendation* or standard* or requirement* or instruction* or guidance* or policies or policy) near/3 (reporting or publishing or good practi#e*))

OR

Keywords: (checklists and guidelines)
