# Supplementary material for: Methodology and reporting quality of reporting guidelines: systematic review
Source: BMC Med Res Methodol. 2015 Sep 22;15:74. doi: 10.1186/s12874-015-0069-z (PMC4579604; doi:10.1186/s12874-015-0069-z)
Supplement: Additional file 2: — Detailed information of the included reporting guidelines. The information of the included reporting guidelines, including publication date, scope, focus, areas, version, and other relevant details. (PDF 161 kb) [file 12874_2015_69_MOESM2_ESM.pdf]

Additional file 2 Detailed information of the included reporting guidelines.

Name of the first author, publication year	Specific website	Scope	Focus	Areas	Version	Consensus	Consensus methods	Referred to the Guidance
Albrecht L, 2013	/	Methods	Systematic review	Knowledge translation	New	Provided	/	/
Debacker M, 2012	/	/	/	Acute Medical Response in Disasters	New	Provided	Modified Delphi technique, Modified nominal group technique	/
Jackson A, 2010	/	/	/	Dose-volume dependencies	New	/	/	/
Gagnier JJ, 2013	Provided	Full text	Case report	General	New	Provided	Consensus meeting	Yes
Weller D, 2012	/	Methods	/	Cancer diagnosis	New	Provided	Consensus meeting, Nominal group technique	/
De Keizer NF, 2012	/	Full text(Conference Paper)	Health Informatics Evaluation	Health Informatics	Update	Provided	Questionnaire survey	/
Gallo V, 2012	Provided	Full text	Observational studies: cohort studies, case-control studies and cross-sectional studies	Molecular Epidemiology	Extension	Provided	Circulating several versions of the statement within the group of developers and an external circle of potential users.	/
Field N, 2014	Provided	Full text	Observational studies: cohort studies, case-control studies and cross-sectional studies	Molecular Epidemiology for Infectious Diseases	Extension	Provided	Circulating several versions of the statement within the group of developers and an external circle of potential users.	/
Janssens AC, 2011	/	Full text	Observational study	Genetic	New	Provided	Consensus meeting	/

Moore CM, 2013	Provided	Full text	Observational study	MRI-targeted Biopsy	New	Provided	RAND/UCLA Appropriateness Method	/
Sun BC, 2012	/	/	/	Emergency Department Syncope Risk Stratification	New	Provided	Delphi	/
Storrow AB,2012	/	/	/	Acute Heart Failure Syndromes	New	Provided	Consensus meeting	/
Chan AW, 2013	Provided	Full text(Protocol)	Prospective clinical trials	General	New	Provided	Delphi, Consensus meeting	Yes
Kearon C, 2010	/	/	Cohort studies	Venous thromboembolism	New	/	/	/
MacPherson H, 2010	Provided	Full text	RCT, case reports.	Acupuncture	Update of extension	Provided	Consensus meeting	/
Salem R, 2011	/	Full text	/	Hepatic Malignancies	New	/	/	/
Black CM, 2010	/	Full text	/	Hepatic Malignancies	New	/	/	/
Harrington NG, 2011	/	/	RCT	Tailored interventions	New	/	/	/
Jackson DL, 2010	/	Full text	/	Rehabilitation Psychology	New	/	/	/
Calvert M, 2013	Provided	Full text	RCT	General	Extension	Provided	Delphi, Consensus meeting	Yes
Piaggio G, 2012	Provided	Full text	RCT	General	Update of extension	Provided	Consensus meeting	/

Robb SL, 2011	/	/	/	Music-based interventions	New	/	/	/
Wu T, 2010	/	Full text	/	Traditional Chinese Medicine	New	/	/	/
Wong G, 2013	Provided	Full text	Realist syntheses	General	New	Provided	Delphi	/
Wong G, 2013	Provided	Full text	Meta-narrative reviews	General	New	Provided	Delphi	/
Welch V, 2012	Provided	Full text	Systematic review	Health equity	Extension	Provided	Consensus meeting	Yes
Beller EM, 2013	/	Abstract	Systematic review	General	Extension	Provided	Delphi, Consensus meeting	/
Melby MK, 2011	/	/	Observational study	Menopause and Aging	New	/	/	/
Wardlaw JM, 2013	/	/	/	Cerebral small vessel disease (SVD)	New	Provided	Delphi	/
de Keizer NF, 2010	/	Full text(conference papers)	Health Informatics Evaluation	Health informatics	Extension	Provided	Web-based survey	/
Ho MY, 2012	/	Abstract	Economic evaluations	Oncology	New	Provided	Consensus meeting	/
Kilkenny C, 2010	/	Full text	Animal study	Animal Research	Extension	Provided	Consensus meeting	Yes
Kottner J, 2011	/	Full text	/	General	New	Provided	Nominal group technique	/
Faggion CM Jr, 2012	/	Full text	In vitro pre-clinical research, RCT	Dental materials and techniques	Extension	/	/	/

Leech NL, 2010	/	Full text	/	Psychology	Extension	/	/	/
Davis JC, 2011	/	/	Economic evaluations	Rheumatology	Extension	/	/	/
Brölmann FE, 2013	/	Full text	RCT	Wound care	New	/	/	/
Cheng C, 2013	/	Full text	RCT	Moxibustion	Extension	Provided	Consensus meeting	/
Dixon WG, 2010	/	Full text	Observational study	Rheumatology	Extension	Provided	Consensus meeting	/
Tong A, 2012	/	Methods and discussion	Qualitative research, SR/MA/HTA	General	New	Provided	Consensus meeting	Yes
Colbert AP, 2011	/	/	/	Complementary and alternative medicine	New	/	/	/
Benchimol EI, 2011	/	Full text	Validation studies	Health administrative	New	Provided	Consensus meeting	/
Eysenbach G, 2011	/	/	RCT	eHealth	Extension	Provided	Delphi	Yes
Moher D, 2010	Provided	Full text	RCT	General	Update	Provided	Delphi ,Consensus meeting	/
Campbell MK, 2012	Provided	Full text	RCT	General	Extension	/	/	/
Husereau D, 2013	/	Full text	Economic evaluations	Health Economic	New	Provided	Modified Delphi	Yes
Gardner IA, 2011	/	Full text	Animal study ,diagnostic accuracy study	Paratuberculosis in ruminants	Extension	Provided	Consensus meeting	/
Xie F, 2013	/	/	Valuation Studies	EQ-5D Valuation	New	/	/	/
Stiles CR, 2010	/	Full text	Prospective clinical trials	Oncology	New	/	/	/

Amor S, 2012	/	Full text	Animal study	Animal models of multiple sclerosis	New	/	/	/
Moore HM, 2011	/	/	/	Biospecimen	New	Provided	Consensus meeting	/
Hoffmann TC, 2014	/	/	/	General	Extension	Provided	Modified Delphi, Consensus meeting	Yes
Smith L, 2010	/	Full text	/	Psychology	New	Provided	Consensus meeting	/
Lang TA, 2013	/	Methods	/	General	New	/	/	/
Kempen JH, 2011	/	Full text	Observational study	Ophthalmology	New	/	/	/
Brouwers MC, 2010	Provided	Full text	Clinical practice guideline	General	Update	Provided	/	/
Meyer E, 2013	/	/	/	Haematology, Oncology	New	Provided	Delphi, Expert opinion	/
Matsumoto M, 2012	/	/	/	Rural and remote health	New	/	/	/
Hooijmans CR, 2010	/	Full text	Animal study	General	New	/	/	/
Hollenbach JA, 2011	/	Methods and Discussion	Observational study	Genetic	Extension	Provided	/	/

/: no relevant information was provided.
